# The potential impact of BCG vaccine supply shortages on global paediatric tuberculosis mortality

**DOI:** 10.1186/s12916-016-0685-4

**Published:** 2016-09-15

**Authors:** Rebecca C. Harris, Peter J. Dodd, Richard G. White

**Affiliations:** 1 TB Modelling Group, TB Centre and Centre for the Mathematical Modelling of Infectious Diseases, Faculty of Epidemiology and Population Health, London School of Hygiene & Tropical Medicine, Keppel Street, London, WC1E 7HT UK; 2 School of Health and Related Research, University of Sheffield, 30 Regent Street, Sheffield, S1 4DA UK

**Keywords:** Bacillus Calmette-Guérin vaccine, Tuberculosis, *Mycobacterium tuberculosis*, Child, Paediatric, Mortality, Shortage, Shortfall, Mathematical model

## Abstract

**Background:**

The Bacillus Calmette-Guérin (BCG) vaccine is provided to over 100 million neonates annually to protect against childhood tuberculosis (TB). Recent BCG manufacturing interruptions highlight global supply risks. We estimated the potential impact of BCG shortfalls on global paediatric (<15 years) TB mortality.

**Methods:**

A static mathematical model was employed to estimate the number of paediatric TB deaths avoided by usual levels of BCG coverage, and potential additional TB deaths in the first 15 years of life due to 1-year BCG supply shortfalls of 6.3 % (as occurred in 2015) to 27.6 % (as anticipated without mitigating action in 2015) assuming no catch-up campaigns.

**Results:**

BCG coverage without shortfalls, estimated at 90 % globally, was estimated to avoid 117,132 (95 % uncertainty range (UR): 5049–306,911) TB deaths globally per birth cohort in the first 15 years of life. An estimated 11,713 (UR: 505–30,691) additional TB deaths would occur in the first 15 years of life per 10 % (26 million dose) annual supply shortfall. A 16.5 million dose (6.3 %) shortfall as reported at the close of 2015, reflecting 84 % global coverage, was estimated as associated with 7433 (95 % UR: 320–19,477) excess TB deaths in the affected cohort in the first 15 years. A possible 24,914 (UR: 1074–65,278) additional deaths were avoided due to prompt shortfall reduction measures in 2015.

**Conclusions:**

BCG shortages could greatly increase paediatric TB mortality. Although rapid action in 2015 minimised BCG shortfalls, avoiding a large number of potential additional deaths, the possible public health impact of even relatively small shortfalls highlights the critical importance of ensuring secure future manufacturing capacity and global BCG supply continuity.

## Background

As one of the earliest vaccines of the World Health Organization’s (WHO) Expanded Program on Immunization established in 1974, Bacillus Calmette-Guérin (BCG) is a cornerstone of childhood vaccination. It is a safe vaccine, used for neonatal vaccination in 84 % of countries and provided to over 100 million neonates annually to protect against childhood tuberculosis (TB) [1].

The vaccine is mostly supplied in 20-dose vials which must be used within 6 hours of opening [2, 3]. Vaccine wastage is therefore common, so annual global BCG demand reaches approximately 260 million doses. Almost half of the global supply is procured through the United Nations International Children’s Emergency Fund (UNICEF). Historically, these volumes have been reliably delivered [4], but sizeable shortfalls began in 2013 due to technical production difficulties. A drop in supply was experienced by both UNICEF and non-UNICEF markets, leading to several, usually self-procuring, countries approaching UNICEF for access to BCG [4]. The shortfall peaked at 22.8 million during 2014, and was 16.5 million at the end of 2015 [5, 6]. Current measures to ensure BCG supply continuity are evidently insufficient.

It has been previously estimated that BCG averts one case of meningeal TB in the first 5 years of life per 3435 vaccinated children and one case of miliary TB for every 9314 vaccinations [7], though this may vary substantially by setting [8]. However, the impact of vaccination on TB mortality and the potential public health consequences of vaccine supply shortages have yet to be explored in the published literature. Therefore, we have used mathematical modelling to estimate the number of excess paediatric (<15 years) TB deaths that could occur with various BCG shortfall scenarios.

## Methods

### Data inputs and assumptions

Data inputs and sources are summarised in Table 1. Our analysis assesses impact at the global level; therefore, where available, data employed are global estimates. Although manufacturing difficulties began in 2013, shortages only began to outweigh mitigation measures in 2014 [9]; therefore, 2013 data were used as the no-shortfall baseline for model parameterisation.

**Table 1:** Data inputs and model assumptions

Variable (symbol)	Point estimate (range)	Source
*Population at risk*		
Annual global BCG-eligible birth cohort (*B*)	130.8 million	[1, 10, 11]
Population vaccinated	HIV-negative	[12]
HIV-negative paediatric TB deaths in 2013 (*Mort*)	80,000 (64,000–97,000)	[19]
*Vaccine deployment*		
Annual BCG demand (*D*)	260 million	[2, 6]
BCG coverage in 2013 (*Cov*)	90 %	[14]
*Vaccine characteristics*		
Vaccine efficacy against TB death (*VE*)	66 % (8–88 %)	[16, 17]
Duration of protection	15 years	[16]
Waning of protection	None	Assumption
Catch-up campaign	None	Assumption

The global BCG-eligible population was defined as the annual birth cohort in countries with a policy of neonatal BCG vaccination, identified as countries reporting BCG coverage to WHO or neonatal vaccination policy in the Global BCG Atlas [1, 10]. This was calculated from the United Nations Population Division 2010–2015 birth estimates [11], assuming equal distribution of births across this period. Although BCG is contraindicated in HIV-positive children [12], the denominator was not adjusted as the proportion of the birth cohort for which BCG is contraindicated due to HIV status is negligible [13].

UNICEF report global demand of 260 million BCG doses per year [2, 6], with vaccination coverage estimated at 90 % in 2013 [14]. BCG dose wastage at baseline (*w*) was estimated at 54.7 %, in line with WHO-UNICEF estimates [2], using current birth cohort (*B*), annual BCG dose demand (*D*), and vaccine coverage $(Cov):w=\frac{\left(D-\left(B\times Cov\right)\right)}{D}\;.$

We explored BCG supply shortfalls of 6.3 %, 10 %, 20 %, 27.6 %, and 100 % of the current 260 million dose global demand; 6.3 % reflects the 16.5 million dose annual supply shortfall at the end of 2015. At the height of the most recent shortages, UNICEF anticipated shortfalls of up to 71.8 million doses in 2015, equivalent to a 27.6 % shortfall on usual levels; 10 % and 20 % are hypothetical shortfalls, of use for estimating impact of other potential scenarios. A complete stockout (100 %) was explored to estimate the impact of each year of BCG vaccination. It was assumed that there would be no catch-up campaign for children missing neonatal vaccination due to shortfalls, as BCG is not recommended for children older than 12 months [15].

A meta-analysis of randomised controlled trials assessing the rate ratio (RR) of TB death in BCG-vaccinated versus BCG-unvaccinated neonates provided a pooled random-effects estimate of 0.34 (95 % confidence interval (CI), 0.12–0.92) [16]. Vaccine efficacy (VE) against TB death is therefore calculated from this rate ratio (VE = 1 – RR) as 66 % (95 % CI, 8–88 %). Similar estimates were made by Colditz et al. [17] (VE 65 %, 95 % CI, 12–86 %) in a 1995 meta-analysis, but are superseded by the Abubakar review [16]. The Mangtani et al. [18] review does not explore vaccine impact on mortality, so cannot be used for parameterisation of this model. The Abubakar et al. [16] review estimates protection against TB death in the overall population, as opposed to against death given disease, therefore this was incorporated in the model as the proportion of the vaccinated population with complete protection against TB death.

Impact was explored over a 15-year time horizon, as this is the WHO-defined reporting age for childhood TB [19]. Data indicate that BCG provides at least 10 years of protection, and there is some indication of protection still at or beyond 15 years [16, 20]. Therefore, it is assumed that those vaccinated are protected for the 15-year model time horizon. There is no clear evidence to parameterise vaccine waning during this period, so no waning is assumed.

As BCG is contraindicated in HIV-positive neonates [12], and more than 90 % of paediatric HIV infections have been attributed to mother-to-child transmission [21], it is assumed that the vast majority of HIV-positive paediatric TB deaths are not BCG-preventable. Therefore, we assume that only HIV-negative paediatric mortality is potentially vaccine preventable (Table 1) [19]. As has been assumed in other BCG modelling studies [8], the annual paediatric TB mortality (‘period mortality’) is assumed to be equivalent to the number of deaths in a 1-year birth cohort over the first 15 years of life (‘cohort mortality’). It is implicit in this assumption that there would be no change in the risk of infection or mortality rates during the 15-year model time horizon.

### Model

A static mathematical model was developed in Microsoft Excel® (2013) to estimate the risk of TB death in the first 15 years of life in unprotected infants, the number of paediatric TB deaths in the first 15 years of life avoided by usual levels of BCG coverage, and the potential number of additional deaths due to 1 year of BCG shortfalls of between 6.3 % and 27.6 % of current global demand.

To estimate the individual-level risk of TB death in the first 15 years of life for children not protected by the vaccine, the number of unprotected children (*n*) in the birth cohort (*B*) was first estimated. Those not protected include unvaccinated children and vaccinated children with insufficient immune response for protection.

1 $$ n=B \times \left(1-Cov\right) + B \times Cov \times \left(1-VE\right) $$

The risk of TB death in the first 15 years of life for unprotected children (*u*_*TB*_) was then calculated by dividing the baseline number of TB deaths (*Mort*) by the number of unprotected children:

2 $$ {u}_{TB}=\frac{Mort}{n} $$

For each of the shortfall scenarios (*s* = 6.3 %, 10 %, 20 %, 27.6 %, 100 %), the number of available doses (*d*_*s*_) and vaccine wastage (*w*) were employed to calculate vaccine coverage (*Cov*_*s*_) of the birth cohort:

3 $$ Co{v}_s=\frac{d_s \times \left(1-w\right)}{B} $$

The number of children unprotected (*n*_*s*_) was calculated for each scenario using *Cov*_*s*_ in equation 1. These were used to estimate the number of additional deaths (*A*_*s*_) for each scenario expected in one birth cohort in the first 15 years of life:

4 $$ {A}_s=\left({u}_{TB} \times {n}_s\right) - Mort $$

### Uncertainty analysis

Uncertainty was estimated by sampling values of paediatric TB mortality and the rate ratio of TB death in vaccinated versus unvaccinated neonates (used to calculate vaccine efficacy) [16, 19]. The underlying distribution of the TB mortality estimates is not published; therefore, a log-normal distribution was generated from the point estimate (80,000) and 95 % range (64,000–97,000) [19]. A log-normal distribution was also employed for the rate ratio, as the published confidence intervals were estimated using the DerSimonian and Laird method [16, 22]. Location and scale parameters for both distributions were estimated using the 2.5 %, 50 % and 97.5 % quantiles in the rriskDistributions package in the statistical programme R [23, 24].

A total of 100,000 parameter sets and model outputs were generated according to the prior-defined probabilities using Oracle® Crystal Ball (release 11.1.2.4.400, Oracle Corporation, USA). Median and 95 % uncertainty ranges for each estimate were calculated from the 100,000 model outputs.

## Results

It is estimated that one year of global BCG vaccination at usual levels of coverage (90 %) averts 117,132 (95 % UR: 5049–306,911) TB deaths during the first 15 years of life of that cohort (Table 2). Therefore, without BCG, the point estimate of annual HIV-negative paediatric TB mortality would be estimated to increase from 80,000 to approximately 197,100. The wide uncertainty ranges derive from the wide confidence intervals around the pooled estimate of the rate ratio of TB death in BCG-vaccinated versus BCG-unvaccinated neonates, as a limited number of studies with relatively small sample sizes from different settings contribute to meta-analyses of this parameter. In our main analysis, it was assumed that the rate ratio of TB death by vaccination status and the global estimate of paediatric TB deaths were uncorrelated and were thus sampled independently. As some correlation may exist, a post-hoc analysis assessing the sensitivity of the results to correlation assumptions was conducted. Even in the most extreme scenario of 100 % correlation between these two variables, uncertainty ranges around the annual number of TB deaths averted at 2013 vaccination levels narrowed only marginally to 5781–244,556.

**Table 2:** Estimated additional paediatric TB deaths in BCG supply shortfall scenarios

Supply shortfall (%)	Number of doses shortfall (million)	Global BCG coverage (%)	Estimated number of additional paediatric TB deaths in a 1 year cohort in the first 15 years of life		Increase in number of deaths (%)
			Median	95 % Uncertainty Range	
6.3	16.5	84	7433	320–19,477	9.3
10	26	81	11,713	505–30,691	14.6
20	52	72	23,426	1010–61,382	29.3
27.6	71.8	65	32,347	1394–84,755	40.4
100	260	0	117,132	5049–306,911	146.4

The hypothetical scenarios explored suggest approximately an additional 11,713 (95 % UR: 505–30,691) paediatric TB deaths in a 1-year cohort in the first 15 years of life per 10 %, or 26 million dose, BCG shortfall. This is a 14.6 % increase in the number of deaths.

A 16.5 million dose (6.3 %) shortfall as reported at the close of 2015, which is reflective of 84 % global coverage, would be estimated to be associated with 7433 (95 % UR: 320–19,477) excess TB deaths in the affected birth cohort in the first 15 years of life (Table 2). A 27.6 % shortfall on global demand was estimated to be associated with 32,347 (95 % UR: 1394–84,755) additional deaths. Therefore, measures to reduce the shortfall from the anticipated 27.6 % to 6.3 % are estimated to have avoided a possible 24,914 (UR: 1074–65,278) additional deaths.

The modelled estimate of the risk of TB death for an unprotected child in the first 15 years of life (*u*_*TB*_) was 0.00151 (95 % UR: 0.00064–0.00299), otherwise expressed as 151 (64–299) per 100,000 children in the first 15 years of life.

## Discussion

Given historical security in BCG supply, the potential public health impact of shortfalls has not previously been quantified. The static mathematical cohort model presented here indicates that even relatively small BCG supply shortfalls could cause sizeable increases in paediatric mortality in the affected cohorts. It is estimated that current coverage of BCG prevents 117,132 (95 % UR: 5049–306,911) paediatric TB deaths in the first 15 years of life per birth cohort, suggesting that an additional 11,713 (95 % UR: 505–30,691) paediatric deaths could be expected per 10 %, or 26 million dose, annual shortfall in BCG supply. Therefore, in the worst case scenario of a 71.8 million dose shortfall anticipated by UNICEF in 2015, up to an additional 32,347 (95 % UR: 1394–84,755) paediatric TB deaths could have been expected in the affected cohort over the 15-year time horizon. However, measures minimising the 2015 shortfall to 16.5 million reduced the estimated number of additional deaths to 7433 (95 % UR: 320–19,477).

UNICEF procures WHO-prequalified (PQ) BCG from five manufacturers. Adjusting quantities awarded to these manufacturers can meet small-scale changes in demand. However, this supply chain is relatively unresponsive to rapid, or large, changes in supply or demand. In recent years, insufficient supply in the non-UNICEF BCG market has inflated demand for UNICEF-procured PQ vaccine from usually self-procuring countries, such as Egypt, Philippines, Pakistan, and South Africa [6, 25]. Thus, the UNICEF market is vulnerable to challenges faced by any manufacturer, not just those supplying PQ vaccine. During the same period, manufacturing shortfalls were also experienced by two PQ vaccine suppliers [4, 6]. These concurrent changes in supply and demand for PQ vaccine far exceeded the responsiveness of the supply system, leading to unmet demand [6].

Rapid and coordinated action from multiple stakeholders to increase supply and to hold buffer stocks at the global rather than national level to allow for prioritisation of limited stocks to high TB-burden countries reduced projected BCG shortages from potentially 71.8 million down to 16.5 million at the end of 2015 [5, 6]. The laudable efforts by multiple stakeholders to minimise BCG shortfalls averted an estimated 24,914 (UR: 1074–65,278) additional paediatric deaths. However, due to the remaining shortfall at the close of 2015, it is estimated that a possible 7433 (95 % UR: 320–19,477) additional paediatric TB deaths could be expected in the first 15 years of life of the birth cohort affected in 2015 alone. Therefore, although rapid action in 2015 minimised shortfalls, avoiding a large number of potential additional deaths, the estimated public health impact of even such a relatively small shortfall highlights the importance of ensuring secure manufacturing capacity to meet global BCG demand.

In the estimates presented, the impact of only one year of shortfall was considered, yet recent shortfalls have already extended over several years. Such shortages affecting multiple birth cohorts would result in even greater public health impact. Resolution of the underlying causes takes time; therefore, strategies to pre-emptively avoid shortfalls are essential to avoid unnecessary childhood illness and deaths from TB. New BCG suppliers have been assessed for pre-qualification to increase availability, but future risks include an anticipated 30 % rise in the BCG weighted average price, the sale of one of the UNICEF suppliers making future production plans uncertain, and on-going supply shortages [5, 6]. It appears unlikely that new manufacturers will enter the market, and there are no known plans for existing suppliers to expand their facilities; therefore, with fixed suppliers and production capacity, mass production of such biologicals will continue to suffer from the small, but very real, risk of production delays.

Reducing vaccine wastage from 20-dose vials could help maximise utilisation of available vaccines. Our model estimates 54.7 % dose wastage, aligning with the WHO/UNICEF-estimated wastage of approximately 50 % [2]. Wastage is particularly prevalent in smaller health centres, where insufficient neonates present for vaccination during the 6-hour vial use window. Economic modelling has demonstrated that clinics vaccinating fewer than seven infants daily would likely benefit from a 10-dose format [26]. It is also believed that availability of smaller vials would be associated with increased willingness to open vials to provide timely vaccination and increased coverage. Therefore, production of both 10- and 20-dose vials for infant vaccination could allow tailoring of supply by setting, reducing wastage, increasing coverage and potentially reducing the risk of shortages.

Another, longer-term solution to BCG supply issues could be implementation of a more time- and cost-efficient manufacturing process. Current BCG supplies are manufactured by the labour-intensive, classic surface growth method, but a more modern, cost-efficient, dispersed, liquid fermentation process currently in use for novel BCG replacement candidates could potentially provide a more consistent product if used in BCG production and could help ensure adequate global supplies (Personal communication, Ann Ginsberg, Aeras, January 20th 2016).

Mathematical models are, by definition, a simplification of reality. Some specific limitations of this approach are considered. As paediatric cases are generally minimally infectious [27], a static cohort model assuming no transmission nor indirect vaccine effects was employed. This limitation may produce an underestimate of the impact of vaccine shortages on TB mortality. The WHO estimate of HIV-negative childhood TB mortality is a key input in this analysis, yet methodology for estimation of paediatric disease burden is evolving, and therefore the size of the estimates may change in coming years. However, such changes do not affect the proportional increase in the number of deaths expected in each scenario, and therefore the impact of shortfalls could be re-estimated in the future using the proportions reported in Table 2. If countries are contributing cases but are excluded from the denominator due to not having a policy of neonatal BCG vaccination, the model could marginally overestimate the effect of the intervention. However, this is expected to be minimal given paediatric TB is generally a sentinel of high transmission, and in such settings BCG is usually offered.

Uncertainty ranges around the modelled estimates are wide due to large confidence intervals around the vaccine efficacy parameter derived from the Abubakar et al. [16] meta-analysis. Factors contributing to this uncertainty include heterogeneity between study settings and the small number of studies included in the meta-analysis (*n* = 5), with relatively low numbers of person years accrued and very few mortality endpoints. All of the studies were conducted between 1933 and 1960. New research could help improve the body of evidence for this parameter, and therefore improve modelled estimates of BCG impact. Placebo controlled trials of BCG are no longer ethical due to lack of equipoise, and retrospective observational designs often suffer from substantial biases, but prospective observational studies with larger sample sizes could potentially contribute to the evidence base for this parameter.

It is possible that some HIV-positive TB mortality may be BCG-preventable, as some healthy HIV-positive neonates with unknown HIV status may be vaccinated, and some children become HIV-infected after receipt of BCG. BCG efficacy against mortality is unknown in these groups, but if BCG were to provide partial protection in these children, the reported impact of BCG shortages would be an underestimate.

Although data are limited, it is possible that BCG protection could wane or the duration of protection could be shorter than the modelled period, and therefore the assumption of 15 years of full protection could produce an overestimate of the impact of shortfalls. Conversely, protection is also likely to continue to prevent deaths beyond 15 years of age, but such longer-term impact was outside the scope of this research question.

There is an increasing body of evidence suggestive of a potentially important non-specific beneficial effect of BCG vaccination against all-cause mortality [28]. Controlled trials and observational evidence from the USA, UK and West African settings have reported point estimates of 25–60 % reduction in all-cause mortality, with impact particularly pronounced in the neonatal period [28, 29]. Therefore, the number of all-cause deaths may also increase with BCG shortfalls, but such non-specific vaccine effects are not accounted for in the model.

It is assumed in the model that neonates missed due to 1-year BCG shortfalls would not receive catch-up vaccination, as WHO does not recommend vaccination above 12 months of age and catch-up campaigns may be difficult to implement [15]. However, for infants under 12 months when supply is restored, catch-up doses may be given to some infants concomitant with other routine Expanded Program on Immunization vaccines, potentially reducing the impact of shortfalls.

UNICEF-reported shortfalls account for demand due to basic country needs and likely some over-procurement to replenish buffer stocks [6]. The scale of over-procurement is unknown and therefore could not be accounted for, but may cause some overestimate of impact in the 2015 scenarios. There are also insufficient data available to quantify the possible mitigating effects of existing buffer stocks in the model. However, in 2014, 28 countries reported BCG stockouts of at least a month at the national level, of which 86 % reported interruption of BCG vaccination services [30]; therefore, country reports clearly support the model assumption that global BCG shortages lead to programmatic vaccine shortages.

This global-level model using a single pooled estimate of vaccine efficacy cannot account for likely heterogeneity in the location of stockouts and possible latitudinal variation in vaccine efficacy [16, 18]. A regional- or country-level model would have been desirable, but estimates of country- or regional-level burden of paediatric TB mortality have not yet been published. When supply shortfalls occur, available UNICEF-procured vaccines are prioritised to high TB burden countries [25, 31]. This could not be accounted for in the model due to the lack of the required mortality burden estimates to develop a country-level model, and there are also no data available on how BCG was distributed. Differential distribution of stock could therefore not be accounted for in this global-level model, and thus may overestimate the impact of shortfalls if high burden countries experience fewer shortfalls. However, this mitigation mechanism does not guarantee supply, especially when the global demand gap is large, as demonstrated in 2014 when six of the 22 WHO high-burden countries reported an interruption of vaccination services [30]. This limitation also highlights the importance of UNICEF-led BCG procurement, as left to market forces alone it is likely that many high-burden countries would struggle to procure sufficient vaccine.

## Conclusion

BCG is a mainstay of neonatal vaccination, yet the risks of supply shortages have not previously been explored. It is clear that even relatively small shortfalls have the potential to cause a considerable number of additional paediatric TB deaths, and the public health risks of larger shortfalls are immense. This is a current issue of global relevance; therefore, continued mobilization to eliminate current shortfalls and development of a secure and responsive mechanism of supply to adequately meet future global demand should be a global public health priority.

## Abbreviations

BCG: Bacillus Calmette-Guérin
CI: Confidence intervals
PQ: Pre-qualified
RR: Rate ratio
TB: Tuberculosis
UNICEF: United Nations International Children’s Emergency Fund
UR: Uncertainty range
VE: Vaccine efficacy
WHO: World Health Organization.
